# Characterization of X Chromosome Inactivation Using Integrated Analysis of Whole-Exome and mRNA Sequencing

**DOI:** 10.1371/journal.pone.0113036

**Published:** 2014-12-12

**Authors:** Szabolcs Szelinger, Ivana Malenica, Jason J. Corneveaux, Ashley L. Siniard, Ahmet A. Kurdoglu, Keri M. Ramsey, Isabelle Schrauwen, Jeffrey M. Trent, Vinodh Narayanan, Matthew J. Huentelman, David W. Craig

**Affiliations:** 1 Center for Rare Childhood Disorders, The Translational Genomics Research Institute, Phoenix, Arizona, United States of America; 2 Molecular and Cellular Biology Interdisciplinary Graduate Program, College of Liberal Arts and Sciences, Arizona State University, Tempe, Arizona, United States of America; 3 Department of Medical Genetics, University of Antwerp, Antwerp, Belgium; 4 Genetic Basis of Human Disease Division, The Translational Genomics Research Institute, Phoenix, Arizona, United States of America; 5 Neurology Research, Barrow Neurological Institute, Phoenix, Arizona, United States of America; University of Bonn, Institute of experimental hematology and transfusion medicine, Germany

## Abstract

In females, X chromosome inactivation (XCI) is an epigenetic, gene dosage compensatory mechanism by inactivation of one copy of X in cells. Random XCI of one of the parental chromosomes results in an approximately equal proportion of cells expressing alleles from either the maternally or paternally inherited active X, and is defined by the XCI ratio. Skewed XCI ratio is suggestive of non-random inactivation, which can play an important role in X-linked genetic conditions. Current methods rely on indirect, semi-quantitative DNA methylation-based assay to estimate XCI ratio. Here we report a direct approach to estimate XCI ratio by integrated, family-trio based whole-exome and mRNA sequencing using phase-by-transmission of alleles coupled with allele-specific expression analysis. We applied this method to *in silico* data and to a clinical patient with mild cognitive impairment but no clear diagnosis or understanding molecular mechanism underlying the phenotype. Simulation showed that phased and unphased heterozygous allele expression can be used to estimate XCI ratio. Segregation analysis of the patient's exome uncovered a *de novo*, interstitial, 1.7 Mb deletion on Xp22.31 that originated on the paternally inherited X and previously been associated with heterogeneous, neurological phenotype. Phased, allelic expression data suggested an 83∶20 moderately skewed XCI that favored the expression of the maternally inherited, cytogenetically normal X and suggested that the deleterious affect of the *de novo* event on the paternal copy may be offset by skewed XCI that favors expression of the wild-type X. This study shows the utility of integrated sequencing approach in XCI ratio estimation.

## Introduction

Diagnosing and uncovering the genetic basis of disease has been revolutionized by whole-exome sequencing (WES), allowing discovery of new disease genes and improving the rate of clinical diagnosis for rare genetic conditions. Indeed, the genetic basis of childhood disorders can be identified in approximately 25% of patients, where successful molecular diagnosis frequently has a major impact on patient management and treatment [1], [2]. Prioritization of candidate variants for the remaining patients remains challenging due mainly to insufficient understanding of the functional consequence of substantial fraction of candidate variants [3]. Large scale functional characterization of genomic variation by simultaneous DNA and RNA sequencing from a patient can reveal genotype-phenotype correlation, can highlight gene expression profile that is associated with the studied genetic condition, and allows immediate evaluation of *in silico* prediction algorithms to the effect genomic variants have on gene expression, alternative splicing, exon usage, gene fusions [4]. In breast and pancreatic cancer integrated analysis of DNA and RNA has been successfully utilized to obtain insight into molecular mechanisms that explain pathogenicity and uncovered potential therapeutic targets to improve patient management [5]–[7]. In addition, RNA sequencing (RNAseq) has been utilized in the context of the affect epigenetic modifications have on gene expression [8], [9]. Integrative analysis of WES and RNAseq data in X-linked disorders may also be informative both in diagnosis and gene discovery for phenotypes emerging due to epigenetic changes such as XCI [10].

In the process of XCI, in females, cells undergo epigenetic inactivation of one of the inherited, parental X chromosomes resulting in consecutive daughter cells expressing one X [11], [12]. The proportion of cells with either parental X as the active is defined by the XCI ratio that ranges from 50∶50 random to 100∶0 completely skewed. Epigenetic analysis of X chromosome in unaffected females indicate that XCI ratio normally distributed in the general population [13]. Although, on the cellular level X-linked alleles are expressed in a dominant fashion due to XCI, in cell populations they show mosaic pattern which can lead to heterogeneous phenotypes in females who are carriers for disease causing, deleterious mutations [14]. In X-linked neurological disease, mode and magnitude of XCI can influence disease severity and outcome [15]. Indeed, case-control studies demonstrate that skewed XCI is common among females who are carriers for X-linked Mental Retardation disorders (XLMR) [16]. XCI may also lead to asymptomatic carrier status by selective advantage of cells expressing the wild-type alleles [17]. One of the difficulties diagnosing females with X-linked diseases and skewed XCI is the broad and overlapping description of clinical phenotype, the limited availability of similar patients, and lack of high-throughput, expression-based methods to estimate XCI [15]. Routine, clinical method to estimate XCI ratio rely on the HUMARA differential DNA methylation assay that targets a polymorphic short tandem repeat (STR) in the human androgen receptor gene (*AR*) [18]. Methylation of this repeat is associated with XCI. Although>90% of females are polymorphic at this site, it provides expression information indirectly from DNA, and, relies on a single locus [13]. There is also conflicting evidence whether DNA methylation can reflect the quantitative expression ratio of active X (Xa) to inactive X (Xi) compared to allele-expression-based methods [19], [20]. Using next-generation sequencing of DNA and RNA simultaneously, we can scan for potential disease causing variations, and at the same time learn about the functional implications of genomic changes with the additional benefit of learning about transmission of alleles and potential imbalance in chromosome X expression due to XCI. By phasing X-linked variant alleles, we can learn about the mode, or parent-of-origin of imbalance, and the magnitude can be estimated from direct measurement of relative expression of chromosome-wide heterozygous alleles.

In this study we present genetic and functional analysis from high-throughput sequencing of WES and RNAseq to both (1) identify potentially pathogenic genetic mutations and (2) identify XCI ratio using phased and unphased allele-specific expression analysis. We show that high-throughput sequencing can be utilized to estimate XCI ratio on simulated data and we apply our approach to a patient with undiagnosed, heterogeneous phenotype. Using family-trio based WES with segregation analysis, we characterized a *de novo*, heterozygous deletion on Xp22.31 as potentially pathogenic, and we identified a moderately skewed XCI ratio from the RNAseq experiment. Integration of exome and expression data revealed that the deletion occurred on the paternal X (Xp), and skewed XCI favored the expression of the cytogenetically normal, maternal X (Xm), suggesting a mechanism for the mild neurological phenotype.

## Materials and Methods

### In Silico Experiment

**Figure 1:**
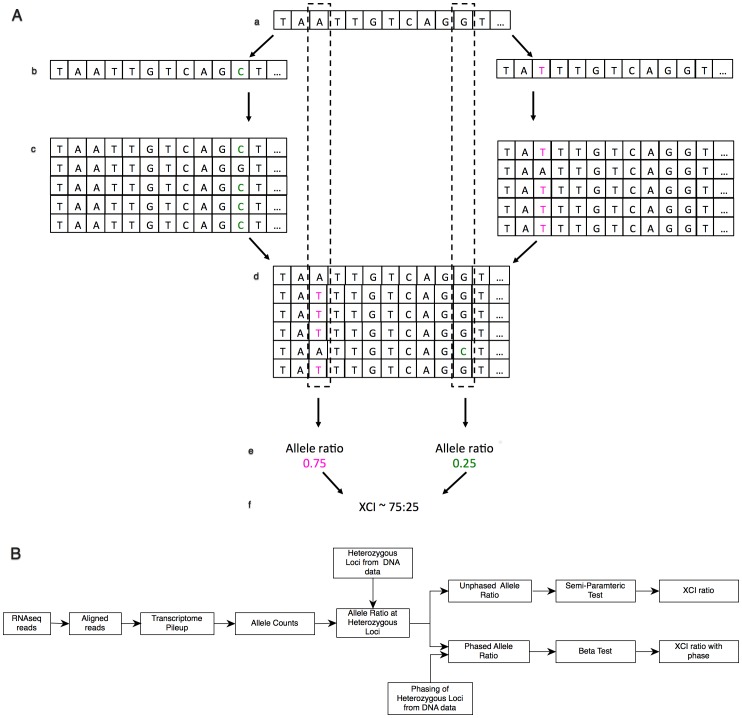
Schematic view of estimation of XCI ratio from read count data. (A) Overview of the simulation study. From a reference transcriptome (a), two haplotypes are simulated with known variant alleles (b). Sequence read simulator generates reads with error attributes using the two haplotypes as reference (c). The reads from both read simulations are merged and aligned back to the original reference (d, dashed lines). Counting the number of reads mapping to each known allele, the allelic ratio of mapped variant alleles can be determined (e). The overall XCI ratio is determined for large number of variants by estimating the mean of the allele ratio distributions of multiple alleles (f). (B) Workflow of XCI estimation from RNAseq experiment using phased and unphased approaches. Essentially, RNAseq reads are aligned followed by obtaining the transcriptome pileup at each sequenced loci. This is followed by counting the number of reads mapping to each allele across the transcriptome. Next, loci are reduced to those that contain heterozygous calls in the genomic DNA and allelic ratio is calculated at each heterozygous locus. If there is no available information on the phase of X-linked alleles at heterozygous loci, the unphased, X-linked allelic ratios are evaluated for their distribution using semi-parametric model and XCI is reported from the parameters of the semi-parametric model. When transmission of alleles can be obtained from DNA data, the phased, X-linked allele ratios are evaluated by the beta distribution and XCI reported from the parameters of the beta model with the phase of XCI.

XCI results in two cell populations in females, one expressing Xm, the other expressing Xp. In theory, the degree of cellular mosaicism due to XCI can be estimated by RNAseq using count-based approach (Fig. 1.A). In this approach, we obtain digital measurement of allele expression from Xm and Xp by counting sequenced reads mapping to each allele, which is directly related to the expression of the chromosome with the allele. On the X chromosome, the allele counts come from either Xa, or Xi, and the ratio of allele frequencies at a heterozygous locus correlates with the overall XCI status of the Xp and Xm chromosomes in the tissue. However, epigenetic modifications, including DNA methylation, cis-, and trans-acting elements, and chromosome strata can influence allele expression at a single locus. Therefore, chromosome-wide heterozygous allele frequency ratio can provide a better estimate of the overall expression of each parental X. In addition, when transmission of alleles can be determined across the X-linked region by allele phasing, in non-random XCI cases, phasing the alleles can identify the parental X that is preferentially inactivated or activated. To evaluate this approach, we simulated RNAseq reads with female, heterozygous genotypes from a pool of known, X chromosome single nucleotide polymorphisms (SNP) in coding regions from the ESP6500 NHLBI Exome Sequencing Project (http://evs.gs.washington.edu/EVS/). The 4996 SNPs were randomly binned in two sets (phased) by rand function of a perl script. In the first set (Alt-P, n = 2520), the alternative allele of the genotype was assigned as paternal, and in the second set (Alt-M, n = 2476), the alternative allele was assigned as the maternal allele. Using *seqtk* FASTA processing tool (https://github.com/lh3/seqtk) the Alt-M and Alt-P alleles were introduced into two separate chromosome X transcriptome fasta files containing known transcripts greater than 500 bp from Homo sapiens.GRCh37.62.gtf. The two modified fasta files were analogous to an X transcriptome with maternal variant alleles and one with paternal variant alleles. Next 10 million, 100 bp paired reads in fastq format were generated, mapping to the two transcriptome files from above (5 million read1 and 5 million read2) using *wgsim* 0.3.1-r13 fastq simulator (https://github.com/lh3/wgsim). Command line options for *wgsim* included zero indel error rate, an outer distance of 150 bp between the paired reads, a uniform Phred quality score of 40 for each base, and a 0.001% base error rate. The combination of these two parental, Alt-M, and Alt-P allele containing fastq files in various ratios followed by mapping them back to the chromosome X reference, and followed by estimation of allelic expression by read count provides the basis for the estimation of XCI ratio. Essentially, after the two modified fastq files with 10 million reads were generated, *seqtk* was used to subsample them randomly, and merge each set into a single fastq file analogous to the reads obtained through RNAseq of an experimental sample. When, for example, XCI ratio of 75∶25 was simulated, 7.5 million correctly paired reads were randomly sampled from Alt-M alleles containing fastq file and 2.5 million were subsampled from Alt-P fastq file and merged. In theory, after alignment and allele count, there would be a 75∶25 allelic imbalance in favor of the Alt-M alleles to an overall chromosome wide 75∶25 ratio since approximately 75% of reads contain alleles from Alt-M. Using this approach, RNAseq reads were simulated for 11 expected X inactivation ratios: completely skewed X inactivation (100∶0), extremely skewed X inactivation (95∶5, 90∶10), moderately skewed X inactivation (85∶15, 80∶20), and random X inactivation (75∶25, 70∶30, 65∶35, 60∶40, 55∶45, 50∶50).

### Estimation of XCI Ratio

Estimation of XCI ratio followed similar steps in both the *in silico* experiment and for the patient (Fig. 1.B). Reads were aligned to human reference genome GRCh37.62 using TopHat2 [21]. Alignment of next generation sequencing data has reference bias that may influence the allelic ratio estimate of SNP alleles. Reduction of bias can be achieved by read alignment to diploid reference incorporating parental genotype information or by reduction of mapping stringency by increasing the number of mismatches allowed in a read for alignment [22], [23]. Therefore five and four mismatches per 100 bp read length were allowed in the *in silico* and clinical experiments, respectively. Allele counts were obtained by generating a chromosome wide pileup with SAMtools mpileup command [24]. Bases with Phred quality score>20 were counted only in the in silico and clinical experiments. Pileup was parsed by an in-house perl script. Next the allelic ratio at each heterozygous locus was calculated by dividing the number of reads mapping to the variant allele with the total number of reads mapping to the locus. After allelic ratio calculation the SNPs were further filtered for quality by following procedure: (1) SNPs within the PAR1 and PAR2 pseudo-autosomal regions were filtered out as they follow autosomal inheritance and can bias XCI ratio [25] (2) Filtered for high confidence variant loci from exome dataset with a genotype filter score of PASS by GATK VariantRecalibrator [26]. (3) Loci without a dbSNP identifier were filtered out (4) Variants with less than 20X coverage were filtered out.

First, phased alleles were used to estimate XCI ratio. Phasing was performed in the *in silico* experiment by assigning the heterozygous variants into their respective Alt-P and Alt-M bin, and by genotype phasing of the trio in the family study as described below. Phasing of X-linked heterozygous variants allows us to evaluate the functional profile of each inherited parental copy. By estimating the parameters (mean, variance) of each copy's allele ratio distribution we can estimate the proportion of cells with Xm or Xp as active and inactive (eg. mean allelic ratio of paternal alleles of 65% and mean allelic ratio of maternal alleles of 35 equals an estimated XCI ratio of 65∶35). To control for over-dispersion of read count data from RNAseq, phased allelic ratios were fitted to the beta distribution to estimate their mean and variance using the fitdistr module of MASS package in R (http://cran.r-project.org/web/packages/MASS/index.html) [27]–[30].

Next, XCI ratio was also estimated without phasing the alleles. When phasing information is unavailable we can lose our ability to define the activity of the parental chromosomes. In this case, the inheritance is unknown and the distribution of allele expression from the two chromosome copies may overlap suggesting similar proportion of cells with one of the parental copies active. However, alleles sampled from the two chromosome copies can have their unique distribution pattern resulting in multi-modal allele distributions. Multi-modal distributions can be understood as a mixture of two or more distributions and thus mixture models based on the expectation maximization (EM) algorithm may be used to estimate the parameters of each component or mode of the distribution. The problem with normal mixture modeling is that the number of components in the data set can greatly affect outcome and advised to account for prior modeling. The semi-parametric (SP) model, however, has no assumptions about the modality or the normality of the data and can also approximate the parameters of each component in a data distribution. In estimation of the inactivation status of the X chromosomes, the mean allelic ratios estimated by the SP model can directly correlate to the proportion of cells carrying the variant alleles. Thus, allelic expression captured in component 1 and 2 of a multi-modal allelic distribution can be thought of as indicators of the proportion of activity of parentally inherited chromosomes in the tissue. The SP method is motivated by the fact that the choice of a parametric family may not always be evident from the distribution of the data, as it is in over-dispersed and heavy-tailed distributions [31]. We applied Bordes et al. stochastic expectation-maximization algorithm for estimating SP model parameters for unphased data [32]. The mean of the estimated component distributions were utilized as the expression status of each inherited chromosomes but were blind to the origin of alleles and applied to define the XCI ratio.

### Family Study

The participating family of Northern European ancestry provided written consent and was enrolled into the Center For Rare Childhood Disorders Program at the Translational Genomics Research Institute (TGEN). The patient was 12 years old at the time of enrollment and verbal assent was obtained from her and documented in writing by the consenting staff person. In addition, written consent for the minor under the age of 18 years was obtained from the parents. All additional participants over 18 years of age provided written consent at the time of enrollment. The study protocol and consent procedure was approved by the Western Institutional Review Board. The primary goal of enrollment is to utilize family-trio based WES in the clinical diagnosis of previously undiagnosed, rare conditions suspected of genetic cause. The female child, now 14 years old had no clinical diagnosis at the time of enrollment, although complex neurobehavioral condition was suspected based on manifesting phenotype of emotional instability, attention deficit, and delays in development and learning. She was born at 38 weeks gestation, and required minimal respiratory assistance. There were early concerns about her development, as she didn't walk until 13–14 months of age. Behavioral problems were noted at age 2, consistent with current phenotypic description above. Treatments with medications for poor attention, impulsivity, repetitive behaviors, and learning difficulties started at age 5. She did not have convulsive seizures, but subtle events consisting of staring, loss of awareness, and tremulousness had been observed. MRIs of the brain were normal; EEG showed right posterior temporal sharp waves. The patient had an older unaffected brother, and her neurological examination was normal showing concrete ability to respond and interpret questions. Previous genetic analysis of genomic DNA from whole blood by array-based comparative genomic hybridization (aCGH) identified a heterozygous deletion between positions 6.4–8.1 Mb on chromosome X. Additionally, HUMARA DNA methylation assay at the *AR* gene identified 85∶15 skewed X inactivation within peripheral blood, providing a hypothesized mechanism for the patient's moderate phenotype. To find possible causal variants that may explain her condition and to validate previous genetic and epigenetic findings whole-exome and RNAseq sequencing was completed on genomic DNA and mRNA isolated from peripheral blood for the mother, father, and patient. Whole blood was collected into EDTA Blood tubes and PAXgene RNA tubes. Genomic DNA was isolated with DNeasy Blood & Tissue Kit (Qiagen, Germantown, MD), and total RNA was isolated from PaxGene RNA tubes using PAXgene Blood miRNA kit (Qiagen, Germantown, MD) following manufacturer's suggested protocol. Exome capture and library preparation was performed with 2 µg of input genomic DNA for each participant using the TruSeq DNA sample preparation kit v2 and the TruSeq Exome Enrichment kit v2 (Illumina, San Diego, CA) following manufacturer's guidelines. The three DNA samples were sequenced as part of a pool of 6 multiplexed libraries on two lanes of a HiSeq2000 v3 flowcell using version 3 of Illumina's multiplexed paired–end sequencing chemistry for 101 bp read length (Illumina, San Diego, CA). RNA library preparation was performed for each family member from 1.5 µg of total RNA using Illumina TruSeq RNA Sample Prep Kit v2 according to manufacturer's instructions (Illumina, San Diego, CA). The three RNA samples were sequenced as part of a multiplexed pool of 4 samples on a single lane of a HiSeq2000 v3 flowcell using version 3 of Illumina's multiplexed paired–end sequencing chemistry for 101 bp read length (Illumina, San Diego, CA).

Binary base calls files were generated by the Illumina HiSeq2000 RTA module during sequencing and were converted to demultiplexed fastq files using CASAVA 1.8.2 (Illumina, San Diego, CA). Quality filtered reads from exome data were aligned to reference genome with BWA 0.6.2-r126 [33]. Binary alignment files were converted and coordinate sorted into the standard BAM format using SAMtools 0.1.18 [24]. Aligned reads were realigned around short insertion and deletions and duplicate reads were filtered using Picard 1.79 (http://picard.sourceforge.net/). This followed aligned base quality recalibration with GATK 2.2 [26]. Flowcell lane level sample BAMs were then merged with Picard 1.79 if samples were sequenced across multiple lanes. Variant calling was done by UnifiedGenotyper and genotype quality recalibrated using VariantRecalibrator as described in the best practice methods of GATK 2.2 [34].

Demultiplexed fastq files obtained from the RNAseq experiment were aligned to human reference genome using ensembl.63.genes.gtf of annotated, known transcripts with TopHat2 [21]. Aligned reads were assembled into transcripts with Cufflinks 2.0.2 using known transcript annotation in ensembl.63.genes.gtf as guide and we used annotated high abundance transcript annotation of ribosomal RNA and mitochondrial genes in an ensembl.63.genes.MASK.gtf. Post transcript assembly, Cufflinks was used to calculate the relative concentration of each annotated transcript by assigning an FPKM value (Fragments Per Kilobase of transcript per Million mapped reads) to each gene and transcript [35].

### Calculation of physical coverage

To determine the boundaries of the interstitial deletion on X, sequence read counts were obtained across X chromosome in a 100 bp sliding window for the mother and child using previously described methods [6]. This script uses the SAMtools package to parse the exome BAM file for the patient and mother [24]. The algorithm uses a sliding window across the selected chromosome in 100 bp length, and for each read mapping within the window finds its mate pair and fills in the gap between the read pairs, then counts this gapped read as one read mapping within the window. This raw read count per 100 bp window is then normalized by dividing the raw read count with the total reads mapping to the sum of sliding windows. Next, the normalized coverage in each window is transformed to log2 scale in both the mother and child and log2 transformed normalized read count is deducted from each other as described here:
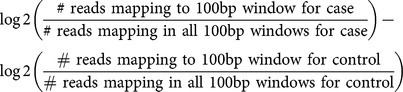



Plotting log2 differences across chromosomes allows detection of large chromosomal deletions and amplifications, where a log2 difference of −1 means a heterozygous deletion in one of the copies.

### Genotype phasing

While any given SNP or indel could be potentially causative towards a disease phenotype, SNPs could also be used as markers for phasing. In this study, we were interested in phasing the deletion and the X inactivation skewing. We refer to the process of phasing as determining the parent-of-origin of a molecular variant (i.e., a heterozygote SNP or mRNA transcript containing a SNP), recognizing that phasing can have broader meanings. In our analyses, we use SNPs as markers to phase a genetic interval or region, where the interval could be a deletion, gene transcript, or chromosome. For example, if the patient is “A/T” for a SNP, the mother is “A/T” and the father is “A/A”, we can determine the “T” allele is from the mother. Larger events can also be phased by examining SNP genotypes contained within the larger event (i.e., a deletion); however, this requires that one recognize that SNP genotypes should be recoded to match their ploidy. For example, males containing a single X chromosome should be understood to be “A” and not “A/A”. Likewise, SNPs within a deletion should be understood to be “T”, rather than “T/T”.

## Results

### Estimation of XCI Ratio from Simulated Data

**Figure 2:**
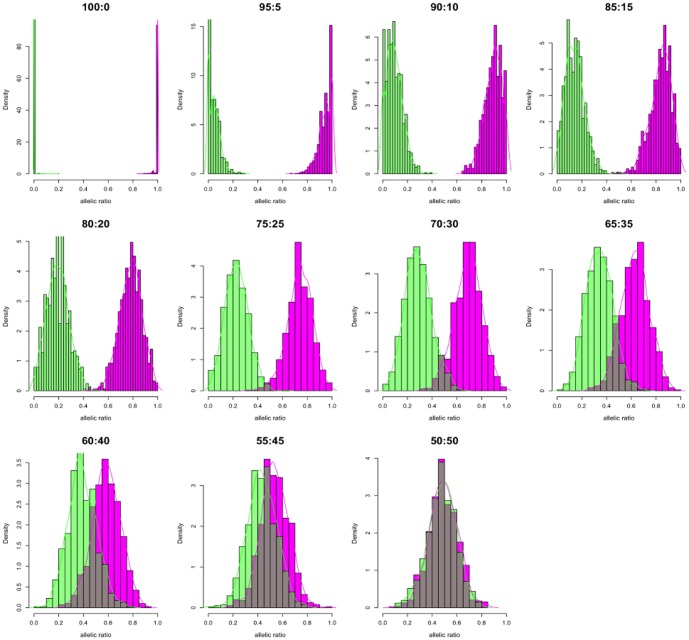
Phasing and distribution of *in silico* allelic ratios. Histograms of showing the allelic ratio distribution after each heterozygous SNP in the in silico data is assigned phase. Each heterozygous SNP allele was covered with at least 20 reads. Alt-M allelic ratios [magenta] and Alt-P allelic ratios [green] in bins of 20. Dark bars indicate SNP ratios that overlap between phased groups. Colored lines are the kernel density estimates of the phased allelic ratio distributions.

**Figure 3:**
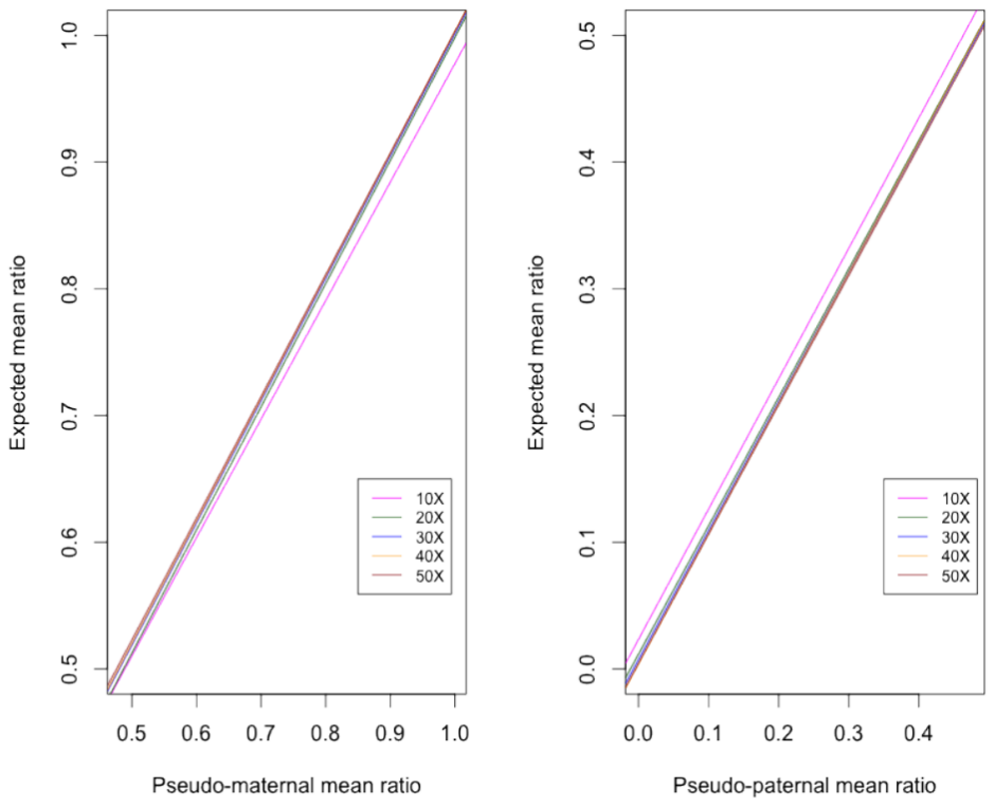
Correlation of expected and observed XCI ratios in terms of sequence coverage. (A) The mean allelic ratio of the Alt-M alleles the *in silico* data to their corresponding expected allelic ratio. Eg. in 70∶30 simulation, Alt maternal alleles have an observed mean allelic ratio of 69.0. (B) The mean allelic ratio of Alt-P alleles from each in silico dataset. Eg. in 70∶30 simulation, Alt-P alleles have an observed allelic ratio of 27.6. Each color indicates the correlation of observed vs. expected ratios at minimum sequence coverage of 10X, 20X, 30X, 40X, and 50X. Pearson correlation coefficient was highest at r>0.9998 above 20X read coverage.

We developed a simulation study for 11 datasets to estimate XCI pattern from paired, RNAseq reads. For each dataset, 4996 loci provided read count information to estimate XCI and on average 1600 SNPs had a minimum read depth of 20. After phasing, the allelic ratios were fitted to the beta distribution and their parameters estimated. The distributions showed increased mono-allelic expression from 50∶50 random to 100∶0 completely skewed XCI (**Fig. 2**). As expected, at 50∶50 XCI ratio the maternal (Alt-M alleles) and paternal (Alt-P alleles) distributions almost completely overlap with their mean ratios at around 0.5 indicating bi-allelic expression and suggesting approximately equal expression of both chromosomes (**Fig. 2** **. 50∶50**). At each expected XCI ratio, the experimental, mean XCI ratios obtained from the beta distributions of the phased allelic ratios showed high concordance with expected XCI (**Table 1**). Although we compensated for read mapping bias by allowing 5 mismatches, our results show some deviation from the expected mean XCI in each dataset. Since our reads were generated against only known transcripts of 500 bp or longer, some sequence homology between transcripts and the other regions of chromosome X may have resulted in read bias affecting allelic ratio estimates. As we shift expected allelic ratios from 50∶50 random toward completely skewed 100∶0, we observed an increased bimodality with the two phases separating into discrete distributions. Coverage analysis indicated high correlation between expected and observed XCI ratios. Although Pearson's correlation was above 0.990 from coverage as low as 10X, correlation coefficient convergence with expected was achieved at>0.999 above 20X suggesting that as low coverage RNAseq experiments may be used for XCI ratio estimation (**Fig. 3**). Unphased allelic ratio distribution followed a similar distribution pattern to phased dataset (**S1 Figure**). Application of SP model to unphased allelic ratios resulted in consistent estimation of expected XCI ratios (**S1 Table**). The mean may be biased by the number of SNP markers available and other factors such as variants in genes that normally escape inactivation. However, our simulation shows that when relatively large number of markers is available, both beta distribution and SP model can consistently estimate the XCI ratio to the expected (**S2 Table**).

**Table 1:** Estimation of XCI Ratio of *in silico* phased SNPs by beta testing.

Expected XCI ratio	Pseudo-maternal	SD	Pseudo-paternal	SD	Observed XCI Ratio
(%)	mean ratio (%)		mean ratio (%)		(%)
100∶0	99.64	1.81	0.06	0.31	99.64: 0.06
95∶5	95.46	11.88	3.91	10.83	95.46: 3.91
90∶10	90.63	15.12	7.96	14.63	90.63: 7.96
85∶15	84.82	14.35	13.11	14.79	84.82: 13.11
80∶20	78.91	12.91	18.01	15.40	78.91: 18.01
75∶25	74.31	12.63	22.59	11.61	74.31: 22.59
70∶30	69.76	14.02	28.87	10.94	69.76: 28.87
65∶35	63.25	11.59	34.11	10.78	63.25: 34.11
60∶40	58.76	11.47	39.15	11.58	58.76: 39.15
55∶45	54.05	11.51	42.88	14.08	54.05: 42.88
50∶50	49.25	11.79	47.84	12.00	49.25: 47.84

XCI  =  X inactivation, SD  =  standard deviation.

### Exome Analysis

WES resulted in an average of 139 million paired reads with average insert size of 249 base pairs [bp] corresponding to an average 14.8 gigabases (Gb) on the HiSeq2000 platform for the trio. After quality filtering, the 121 million average reads were aligned to reference with an 88% alignment rate. Approximately 97% of target regions had a mean base coverage of 10X (**S3 Table**). Joint variant calling identified 85,708 single nucleotide variants (SNVs) and short indels in with 85.96% of calls in dbSNP135 (http://www.ncbi.nlm.nih.gov/projects/SNP/). Functional evaluation of calls identified 42,192 [46%] missense, 344 non-sense (0.38%), and 48,373 (53%) silent variations. Transition/transversion ratio was 2.31 for all calls, and 2.447 for dbSNP variants. We applied various filtering approaches described elsewhere, but extensive search within Clinvar (http://www.ncbi.nlm.nih.gov/clinvar/), The Human Gene Mutation Database (http://www.hgmd.org/), and OMIM (http://www.ncbi.nlm.nih.gov/omim/) did not identify any unambiguous genetic variants that likely caused or contributed to the child's phenotype [3].

### Characterization and Phasing of Xp22.31 Deletion

**Figure 4:**
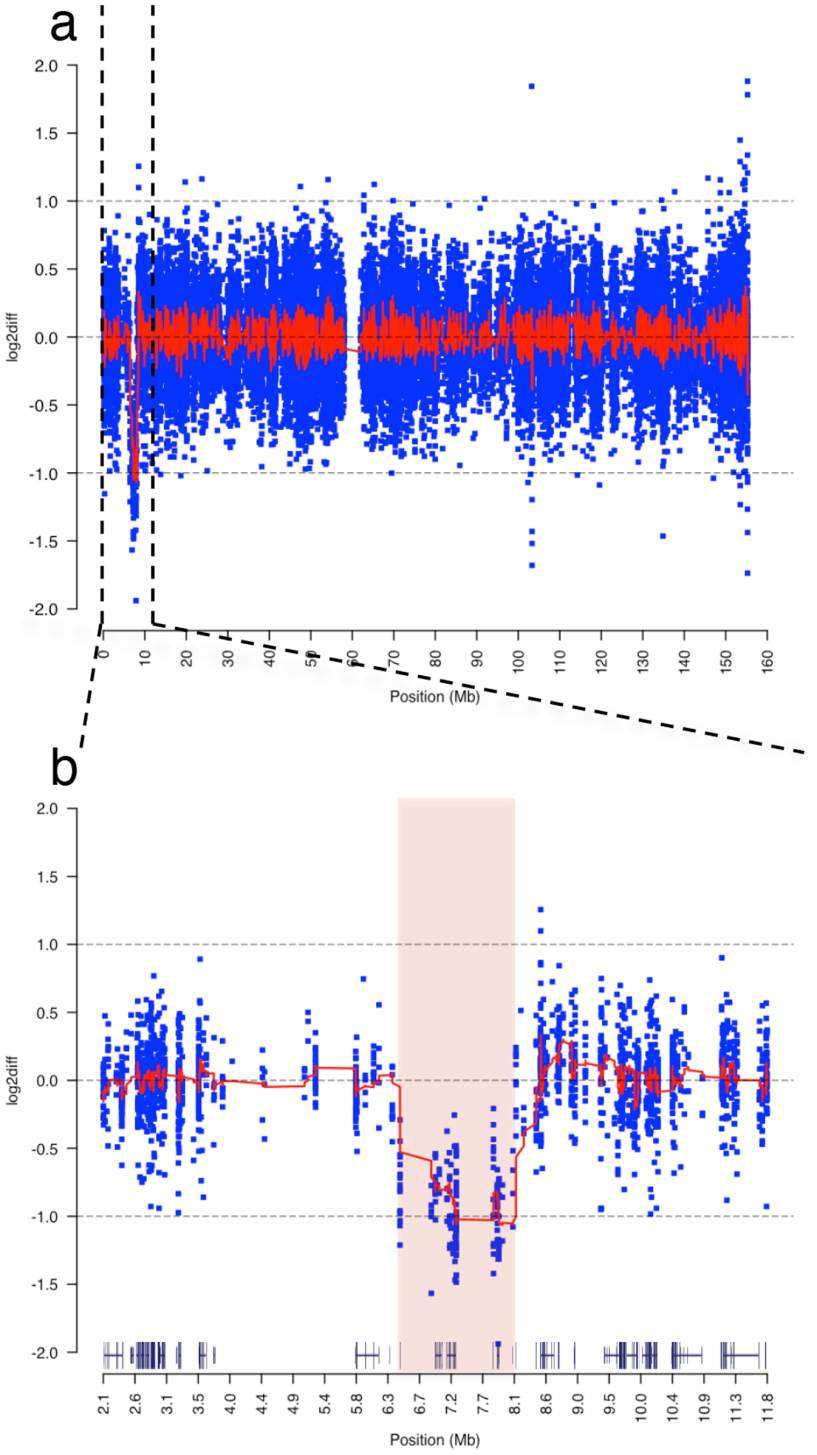
Characterization of *de novo*, interstitial, heterozygous deletion on Xp22.31. (a) Chromosomal view of log2 coverage difference between affected child and mother obtained by WES. The log2 difference of normalized read coverage between affected child and mother is shown on the y axis, with each blue dot indicating log2 difference in normalized sequence coverage in a 100 bp window. The red line across the chromosome is the mean log2 differences across a sliding window of 25. A large deletion on chromosome X is recognizable in the child indicated by drop in log2 difference to −1 between 0–10Mbase. (b) Zoomed in view of reduced sequence read coverage between 6.4–8.1Mbase of the short arm of the chromosome. The pink shaded area indicates the deletion breakpoints predicted by aCGH analysis that overlaps with deletion seen by the exome coverage analysis. Gene tracks above the x-axis were obtained from UCSC Genome Browser and contain the deleted genes *VCX3A*, *HDHD1*, *STS*, *VCX*, *PNPLA4* genes and *MI4767* microRNA genes.

**Figure 5:**
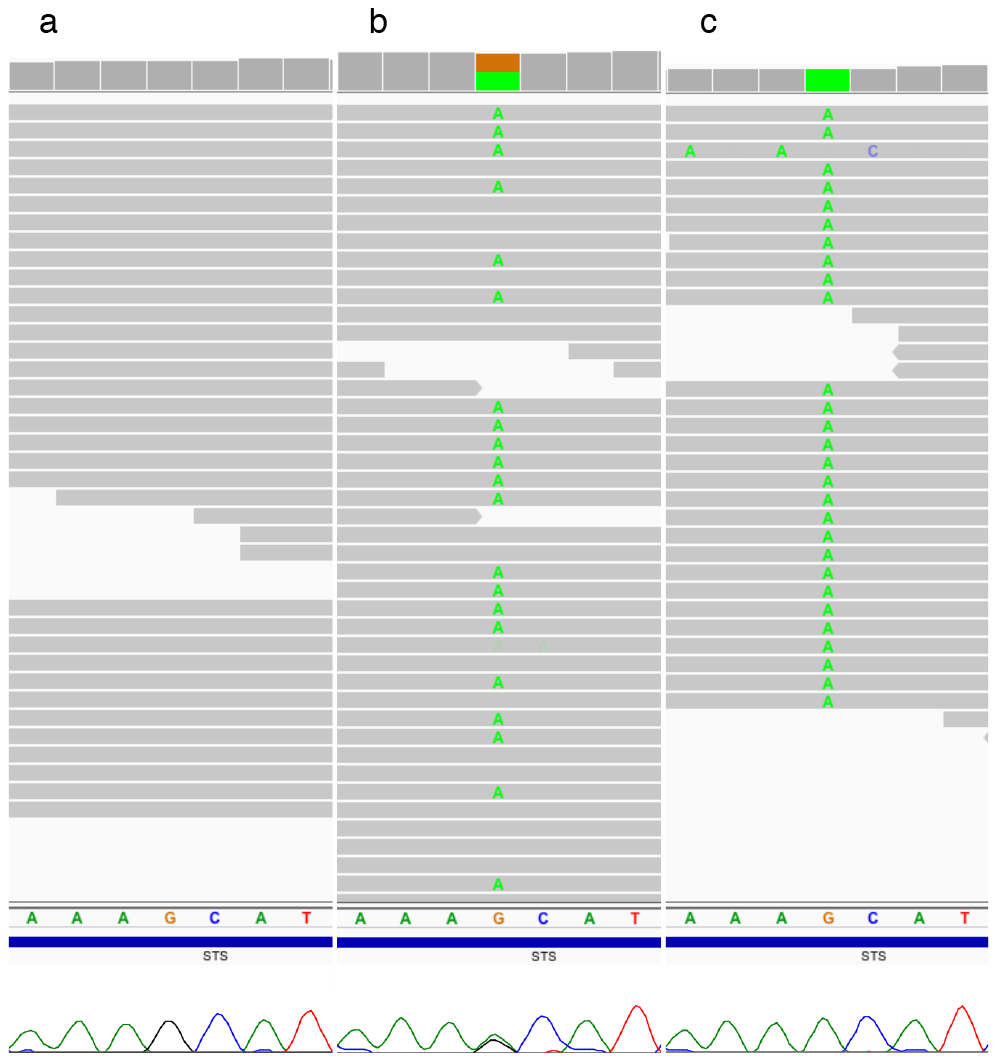
Determining phase of rs5933863. Next-generation sequencing traces visualized using the Integrated Genomic Viewer (IGV) and below them the corresponding Sanger traces of rs5933863 G>A alleles in the *STS* gene that helped determine phase and origin of the 1.7 Mb deletion on chromosome X [54]. Patient's IGV and Sanger traces (a) indicate that she is either homozygous G/G or hemizygous “G” genotype at this position. The mother's (b) and the father's (c) traces indicate that they are “G/A” and “A” genotype, respectively.

Absence of candidate rare variants focused our attention to the previously identified interstitial deletion on Xp22.31. We compared log2 normalized physical coverage of the daughter's exome to the log2 normalized coverage of the mother's [see methods], and observed those regions where the ratio fell below the threshold coverage of −1. Comparative analysis identified the deletion as heterozygous at Xp22.31 with breakpoints at 6,451,600 and 8,095,100, respectively (**Fig. 4**). Similar comparison to the father's exome indicated that father was hemizygous for this region; therefore the deletion occurred *de novo*. The distal breakpoint is approximately 50 bp upstream of the variable charged X-linked 3A gene [*VCX3A*] and the proximal breakpoint resides within the first 100 bp of miR-651, a microRNA gene with no known biological function. The deletion encompasses 1,643,501 bp harboring five genes and two microRNA genes (**S4 Table**). This region was in concordance with the aCGH. The deletion was phased to Xp based on rs5933863, at X∶7,270,694 G>A in the 3′ un-translated UTR region of the *STS* gene (NM_000351). The affected child's genotype was homozygous G/G, the mother's was heterozygous G/A, and the father's was homozygous alternative A/A. Recoding based on anticipated ploidy, the child's genotype is “G”, the mother remains “G/A”, and the father with a single X chromosome is recoded “A”. Principles of X-linked inheritance dictate that the child must have a heterozygous genotype G/A at this position. Since she is missing the paternal allele A and has an apparent genotype of “G”, there is evidence that the region containing this SNP on Xp was deleted resulting in an out-of-phase genotype (**S5 Table**). This out-of-phase coding SNP was validated by Sanger method in the trio Fig. 5.

### Estimation of XCI Ratio from RNAseq experiment

**Figure 6:**
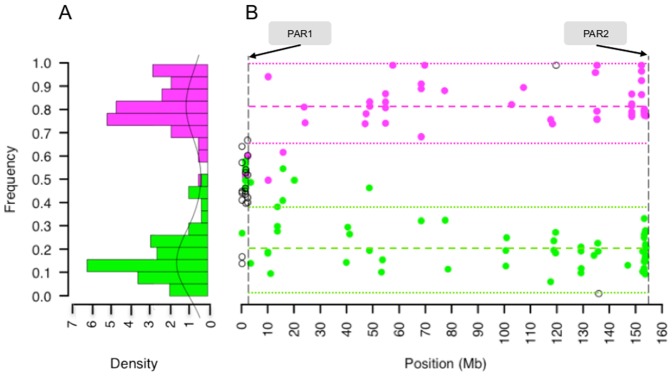
Phased allelic expression on chromosome X. (A) Allelic ratio of heterozygous SNPs show bimodal distribution of the expressed maternal (magenta dots, n = 37) and paternal (green dots, n = 44) alleles indicated biased expression of the inherited chromosomes. (B) Chromosome-wide allele frequency of the phased alleles from RNAseq indicate that overall, maternal X has a preferential expression in the patient with mean ratio across X of 0.82.7±0.083 (dashed magenta line), compared to paternal alleles of 0.20.3±0.095 (green dashed line). Biased expression in favor of the maternally inherited alleles is preserved across the entire length of the chromosome. However, alleles within genes that potentially escape X inactivation can show bi-allelic expression as defined by an allelic ratio 2SD outside the mean of the phased allele ratios (colored, dotted lines). Essentially all high quality heterozygous SNPs with a minimum of 20X coverage could be phased based on transmission of alleles within the X-linked region. SNPs where transmission of alleles could not be determined (clear circle) lie predominantly in the pseudoautosomal region (PAR1) except two Mendelian errors.

Sequencing the patient's mRNA resulted in an average of 116 million paired reads per sample mapping to human reference genome (**S6 Table**). From the exome variant call set 1,729 single nucleotide variants including indels mapped to chromosome X, of which 901 were called heterozygous in the affected child. 374 calls were heterozygous SNPs within transcripts, and 325 were X-linked, outside PAR1 and PAR2 regions [25]. 226 variants were high quality with score PASS by GATK Variant Recalibration. Next we selected variants that were previously documented in dbSNP build 135. A total of 83 SNPs were covered with at least 20 reads. 37 phased to Xm and 44 to Xp, and two Mendelian errors. The 37 Xm alleles were from 23 genes, with 19 genes with a single heterozygous expressed variant and four had more than two heterozygous expressed variants. The 44 Xp alleles were from 31 genes, and 22 of them had a single heterozygous variant expressed and 9 had more than one heterozygous variant. The allele ratio distribution indicated bimodal distribution showing lower expression of paternally inherited heterozygous SNPs (**Fig. 6**). The XCI ratio estimated from phased alleles was 82.7∶20.3 (approximately 83∶20), and from the unphased allelic data was 82.2∶19.2 (approximately 82∶19), consistent with moderately skewed X inactivation with a ratio of 85∶15 obtained by the HUMARA methylation assay. The integration of phase information had minimal affect to final estimate indicating the power of the SP model. In addition to the patient, we estimated XCI ratio in 4 additional female individuals from our clinical sequencing center (**S2 Figure**). In each case XCI was estimated by our RNAseq approach and the HUMARA assay. A single case was uninformative for the HUMARA, which can be due to homozygosity at the methylation sensitive repeat sequence of the *AR* locus (**S34 in** **S2 Figure**). In 3 out of the 5 cases (60%), the HUMARA method suggested moderately skewed XCI ratio (>80∶20) (**S14, S18, S23 in** **S2 Figure**). However, expression analysis supported strong correlation between the three methods only in the clinical case of this report where skewed XCI was estimated by all three methods (**S18 in** **S2 Figure**). In three of the remaining four cases skewed XCI was not supported by the RNAseq analysis (**S14, S23, S34 in** **S2 Figure**). In a single case all three methods predicted random XCI ratio (**S11 in** **S2 Figure**). In general there is a high concordance between the three approaches with the beta and the SP methods have the highest concordance (Pearson's r  = 0.99), but these approaches have weaker correlation with HUMARA (SP Pearson's r = 0.84, beta Pearson's r = 0.80). In general, we see a lower XCI ratio estimated by allele expression analysis than by HUMARA. Estimates of XCI ratio may be biased by reference bias in read mapping, insufficient coverage at heterozygous loci, and by heterogeneous gene expression due to methylation and cis-acting regulatory mechanisms.

### Identification of Genes that Escape X inactivation

Phased, allele-specific expression analysis highlighted a number of variants in genes that may escape inactivation. Escape of X inactivation results in bi-allelic expression of genes from Xa and Xi in the same cell and can contribute to phenotypic variability in females who are carriers of X-linked disease [36]. Therefore a catalogue of escape genes in clinical evaluation may contribute to the better understanding of clinical symptoms and may offer treatment options. We identified escape genes in the patient by examining 325 heterozygous loci across X and the deviation of their allelic ratio from the mean allelic ratio of each phased distribution. We defined a candidate escape gene by having a heterozygous SNP with an allelic ratio two standard deviations (2SD) outside the mean allelic ratio of the chromosome-wide allelic distribution and showing bi-allelic expression. Bi-allelic expression was defined as allelic ratio between 0.1 and 0.9. Therefore if a paternally inherited variant had an allelic ratio of 0.49 and the mean allele ratio of the chromosome-wide paternal alleles was 0.203 with a standard deviation of 0.09, that variant allele ratio was greater then 2SD from the mean, thus was bi-allelic expressed. Of the 325 X-linked heterozygous alleles 15 showed bi-allelic expression in 12 genes, but 7 variants were considered false positive due to low read coverage (<7X) (**S7 Table**) [37]. Comparison of the sufficiently covered variant loci to chromosome wide XCI screens in hybrid cell lines and fibroblast indicated that in 4 of the 6 escape genes, XCI status was consistent with previous assignments of genes as escaping from X chromosome inactivation using both hybrid cell line and fibroblast data. Protein Convertase 1 Inhibitor (*PCSK1N*) and Plexin A3 (*PLXNA3*) both suggest escape status in the patient, and were previously reported as subject of XCI [36]. *PCSK1N* and its associated propeptide may have a role in body weight and behavior in mice, and Plexin A3 is a co-receptor of the axon guidance receptor, Neurophilin-2 (*NRP2*) but their dosage affect due to XCI remain to be elucidated [38], [39]. The distribution of genes that are shown to escape XCI was consistent with the regions that contain the highest density of escape genes, and were mostly located on the short arm of chromosome X [40].

## Discussion

In this study we applied integrated WES and RNAseq to simultaneously evaluate the functional effect of coding variations in the process of clinical diagnosis. Although previous clinical testing suggested a mechanism for the patient's disease, with the combined analysis of the trio exome and the patient's RNA expression that we are now able to hypothesize a mechanism for the observed phenotype. Variant filtration approaches after trio WES did not result in the identification of strong candidate causal variations. Although there was suggestive evidence from the aCGH that the disease pathology may be related to a heterozygous deletion on Xp22.31, it was only with incorporation of SNP phasing and comparative analysis of sequenced reads that we were able to determine that the deletion occurred *de novo*. Genes associated with neurological dysfunction including a number of variable-charge X-linked genes lie within the deletion (*VCX*, *VCX3A*) [41]. Although we were not able to detect lymphocyte expression of any of the *VCX* genes, there is suggestive evidence these genes have roles in cognitive function. *VCX3A* overexpression in rat hippocampal neurons increase neurite outgrowth that may positively influence synaptic plasticity [40]. Furthermore, some males who are hemizygous for a recurrent Xp22.31 deletion and have X-linked ichthyosis (OMIM 308100) also demonstrate mental retardation [42]. This region appears to be a hotspot for copy number changes, complex duplications, and triplications, suggesting that the instability of this region may contribute to disease risk [43]. The inherent limitation of our approach is that our resolution to define the exact genomic content of the deletion is reduced by exome sequencing and can only be circumvented with whole-genome sequencing approaches.

Phased and unphased allele-specific expression in the patient was concordant with the HUMARA assay and indicated moderately skewed XCI. The contribution of skewed XCI to her condition is not clear, although the phased XCI ratio allows us to develop a hypothesis for the molecular mechanism that underlies her condition. One could hypothesize that random XCI in the patient and potential dominant negative affect of the deletion would result in a severe neurological condition. However, females who are carriers for deleterious chromosomal mutations may not present clinical symptoms due to selective advantage and preferential expression of the normal X [16], [44]. These females are usually heterozygous for an X-linked deleterious allele and have skewed XCI. The patient has skewed XCI and is heterozygous for the deletion but showing some mild neurological condition, suggesting that the preferential expression of the cytogenetically normal X may be compensating for the deleterious affect of the deletion. While insufficient cases have been reported to provide statistical significance, females who were diagnosed with Xp22.31 microduplication and preferentially silenced the X with the microduplication had normal phenotype while those who preferentially express the X with the microduplication had intellectual disability [45]. It is plausible that loss of a chromosome copy at Xp22.31 has different clinical manifestation than copy gain. Therefore the contribution of Xp22.31 rearrangements to neurological dysfunction need further study. For the patient sequenced in this study, our data are consistent with a model that the preferential expression of the cytogenetically normal, maternal X may have contributed to her mild cognitive phenotype.

Our ability to uncover molecular mechanisms by DNA and RNAseq in patient's surrogate tissue (peripheral blood) that may correlate with phenotype in the central nervous system argues for potential benefit in clinical diagnostic cases that remain unresolved. This is supported by a number of studies that find a strong correlation in gene expression profile in blood with affected status in such diseases as Parkinson's Disease and Huntington's Disease [46], [47]. Previous studies evaluating the methylation status of X-linked genes and overall XCI patterns across various tissues show that XCI is concordant between tissues, including blood and brain [48], [49]. However, these studies were performed in females with no known neurological condition and showed that variable XCI status exists in about 12% of X-linked genes and variance between tissues increases with age. Studies in Rett syndrome and XCI in mice show some evidence that deleterious alleles lead to preferential silencing of the mutant X in brain tissue, but their correlation with blood has not been well characterized [50]. In females with Rett Syndrome there is evidence that skewed XCI correlates with disease, however correlation between blood and brain XCI pattern was low in a small sample set. Therefore the use of whole blood to predict XCI patterns in the brain and their correlation to disease susceptibility remains to be elucidated.

Our simulation proposed an approach to estimate XCI ratio using chromosome-wide SNP expression and found that phased and unphased SNPs can equally estimate the ratio with both beta and SP model. Even if research and clinical sequencing application will be limited in sequence coverage, our method is able to predict XCI at high concordance with expected as low as 10X coverage. Our method also allowed for base error rate therefore providing a more realistic sequence data. Our approach based on read count, and relative ratio estimation of variant alleles, can be applied to other sequencing platforms and to other expressed regions of the genome that are targeted by RNAseq. Principles of skewed expression demonstrated in this study could be relevant to imprinted portions of autosomes and therefore applicable to disorders like Prader-Willi and Angelman syndromes [51]. Skewed expression of autosomal heterozygous alleles can be markers for imprinted regions, and may uncover cis-regulatory elements.

Although, in our small dataset, XCI estimation from RNAseq analysis was not fully concordant with the methylation assay, direct measurement of allele expression may provide a better estimate of the true cellular activity of each inherited chromosome copies. HUMARA assay targets a single genomic locus and relies on the methylation of a repeat sequence targeted by methylation sensitive restriction enzyme. Deletions, copy number changes, homozygosity at the AR locus, biases due to enzymatic and PCR inefficiency, hypo-methylation of restriction enzyme target, difficulties associated with data interpretation, and the challenges associated with the amplification of repeat regions may influence assay results.[20].

Our approach is dependent on the accuracy and sensitivity of multiple SNP markers expressed in the X-linked region. There is heterogeneity in the regulation of X-linked gene expression by epigenetic mechanisms, therefore, sampling alleles from multiple genes with various expression levels to infer XCI ratio may be inconsistent with previous methods but excluding alleles from genes that escape XCI can provide an inaccurate picture of the X chromosome activity, and molecular characteristics of the tissue source [36]. Therefore, we did not filter out alleles from genes that were previously reported to escape XCI. This may have contributed to an overall lower XCI ratio estimates by RNAseq compared to the methylation assay. In addition, methylation based assessment of XCI may not be concordant with expression based methods due to differences in assays and applied analytical methods. Challenges in RNAseq experiments include technical and analytical variability that may affect XCI ratio therefore transcription-based validation assays may be useful to improve our approach [36], [52], [53]. The use of direct expression analysis of multiple SNP markers may also increase our power to accurately estimate XCI, providing a basis to improve our definition of clinically significant XCI ratio boundaries. However a more systematic screening of XCI by RNAseq across a series of X-linked disorders in females may greatly enhance our understanding of the underlying cause of phenotypic variability.

In conclusion, WES identified a deleterious deletion on Xp22.31 that is in a hotspot for chromosomal rearrangements and associated with a number of neurological conditions. In addition, using allele-specific expression analysis from RNAseq we were able to define XCI ratio in simulated and experimental data. Although the number of individuals reported, and the number of heterozygous alleles in the X-linked region may be small, both the SP and beta models could reliably estimate XCI from RNAseq data. The benefit of the SP model is that parental sequencing and genotype phasing is not necessary to estimate XCI, it compares well to XCI based on allele phasing, and can be applied to individuals only. The combined genomic and functional data allowed formulating hypothesis for the molecular mechanism for the patient's symptoms, which can provide a basis for further clinical studies and patient management. However, extensive functional analysis is required to assess if our hypothesis based on sequencing blood RNA can be applied to a neurological condition. Finally, our study also represents an application of high-throughput sequencing methods and their simultaneous utilization to study epigenetic mechanisms in the clinical settings and how they contribute to genetic basis of a heterogeneous disease. With the rapid decrease in sequencing costs and improved analytical methods, comprehensive, integrative sequencing approaches will likely be used more in the future and may replace traditional methods that may be uninformative due to atypical disease phenotype, low-throughput, high costs and invasiveness.

## Supporting Information

S1 Figure **Un-phased allelic ratio distributions.** Histograms showing the allelic ratio distribution after each heterozygous SNP in the in silico experiment when phase is not assigned. Each heterozygous SNP had to be covered with at least 20 reads. Black lines indicate the Gaussian kernel density of unphased allelic ratio distributions. Similar to phased experiments, the shift of distributions from unimodality in random XCI (50∶50) toward bi-modality as XCI becomes more skewed towards 100∶0 complete skewing.
(TIF)

S2 Figure **Estimation of XCI ratio by allelic expression and DNA methylation assays.** XCI estimated in five female patients. The x-axis indicates the approach (Beta =  beta distribution of phased allelic expression, Hum =  HUMARA DNA methylation assay, SP =  semi-parametric method of unphased allelic expression). The y-axis indicates the XCI ratio (eg. S11 XCI ratio by Hum  = 75∶25). XCI ratio estimated by fitting allele ratios to the beta distribution can provide information about parental bias in XCI ratio as in the patient (S18) has 82.7∶20.3 biased XCI that favors the expression of Xm (magenta). The ratio of allele expression from the maternal chromosome to the allele expression from the paternal chromosome (blue) gives the XCI ratio. In S18, using the beta model, we were able to determine that moderately skewed XCI ratio favored the expression of Xm compared to Xp. We had no phase information on the *AR* locus for the HUMARA assay, thus phase of XCI could not be determined. Due to homozygosity at the *AR* locus, S34 was uninformative for HUMARA, underlying the utility of RNAseq in XCI estimation. The SP method does not consider allele phase to estimate the parameters of allele distributions, so phase of XCI could not be determined. RNAseq estimates random XCI (<80∶20) in S14 and S23 compared to moderately skewed XCI (>80∶20) by HUMAR. S18 and S11 show complete concordance between the three methods. There is no clear trend that would indicate a higher likelihood of biased inactivation of either parental chromosome.
(TIF)

S1 Table **Estimated XCI ratio of unphased data by SP method across 11 in silico experiments.**
(TIF)

S2 Table **Number of heterozygous variants to estimate XCI after filtering for coverage.**
(TIF)

S3 Table **Summary metrics of Exome Sequencing.**
(TIF)

S4 Table **Genes within 6,4-8,1 Mb interstitial deletion.**
(TIF)

S5 Table **Genotype phase of X-linked SNPs within the 6,4-8,1 Mb interstitial deletion.**
(TIF)

S6 Table **Summary metrics of RNA Sequencing.**
(TIF)

S7 Table **Escape of XCI status.** Phased allelic ratios for SNP variants outside 2SD from the mean of phased allelic ratio distributions. Escape status was compared to XCI status observed in Carrel at al. [36] hybrid cell line and fibroblast cell line Xi expression. “Escape”  =  Gene escapes XCI. Gene expression on Xi seen ≥78% of cell hybrids and fibroblasts. “Heterogeneous”  =  Gene expression on Xi varies between 22–78% cell hybrid and fibroblast cells. “Subject”  =  Gene subject to XCI. Gene expression on Xi is seen in ≤22% of cell hybrid and fibroblast data. “-“  =  Not reported.
(TIF)
