# Supplementary figures and images for: Characterization of X Chromosome Inactivation Using Integrated Analysis of Whole-Exome and mRNA Sequencing
Source: PLoS One. 2014 Dec 12;9(12):e113036. doi: 10.1371/journal.pone.0113036 (PMC4264736; doi:10.1371/journal.pone.0113036)

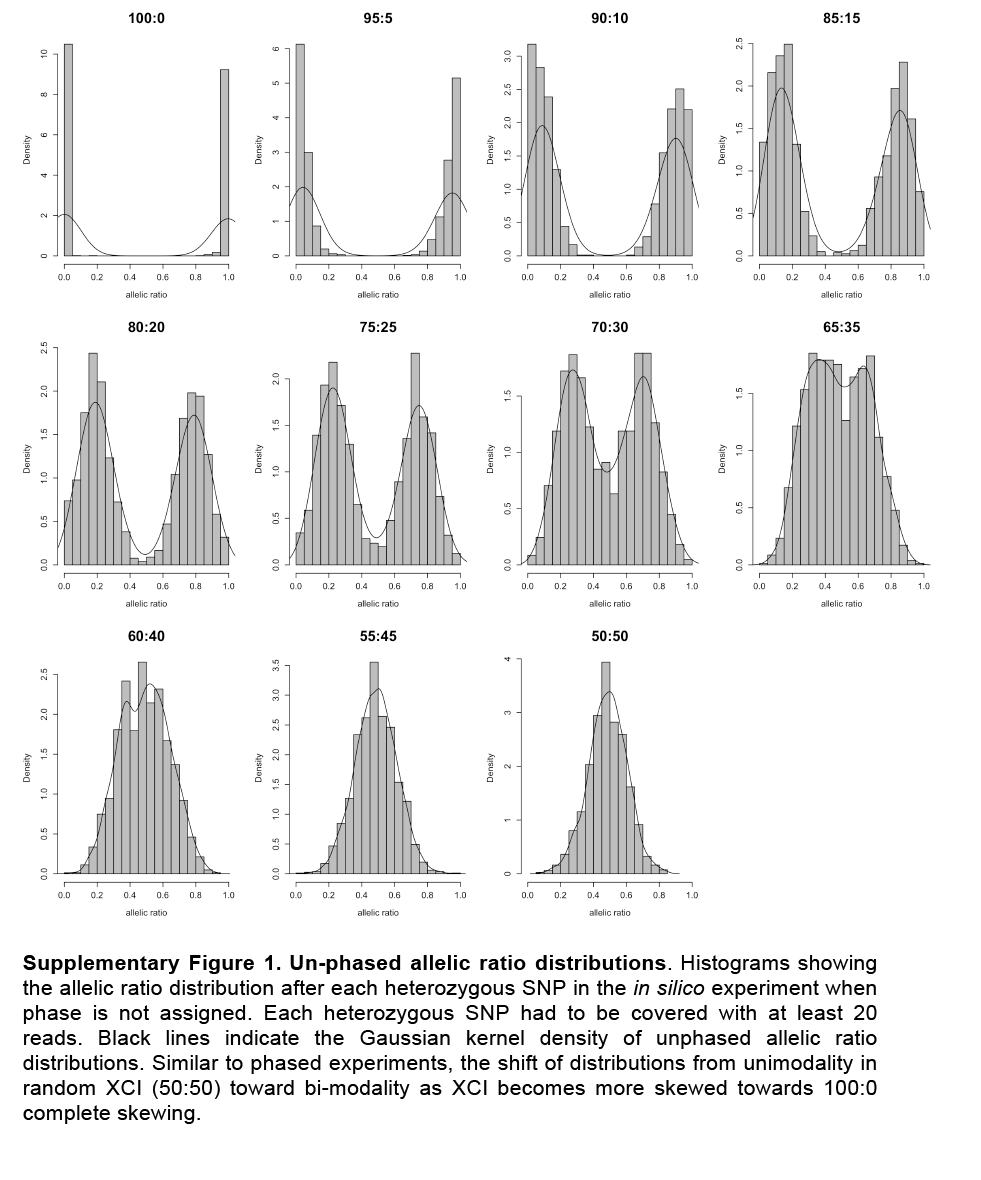

Supplement: S1 Figure — Un-phased allelic ratio distributions. Histograms showing the allelic ratio distribution after each heterozygous SNP in the in silico experiment when phase is not assigned. Each heterozygous SNP had to be covered with at least 20 reads. Black lines indicate the Gaussian kernel density of unphased allelic ratio distributions. Similar to phased experiments, the shift of distributions from unimodality in random XCI (50∶50) toward bi-modality as XCI becomes more skewed towards 100∶0 complete skewing. (TIF) [file pone.0113036.s001.tif]

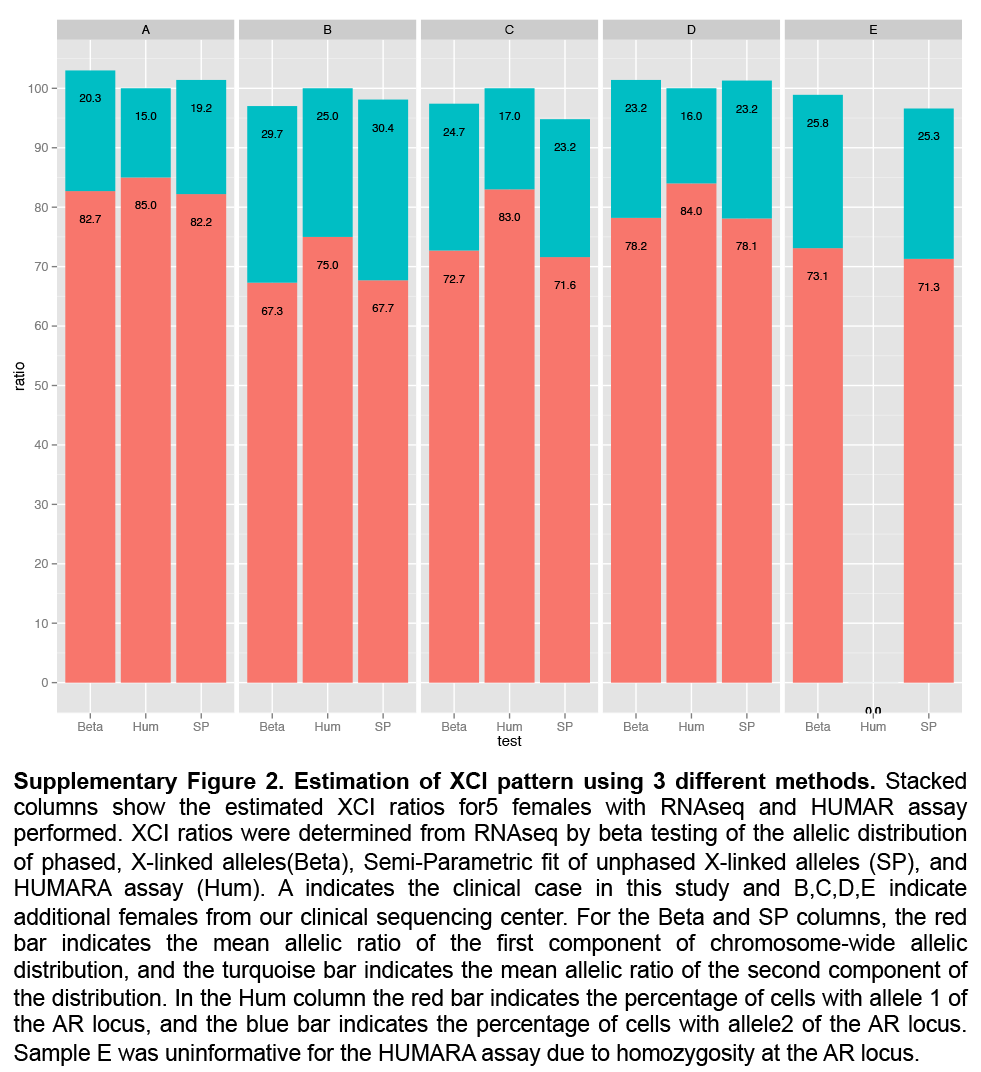

Supplement: S2 Figure — Estimation of XCI ratio by allelic expression and DNA methylation assays. XCI estimated in five female patients. The x-axis indicates the approach (Beta = beta distribution of phased allelic expression, Hum = HUMARA DNA methylation assay, SP = semi-parametric method of unphased allelic expression). The y-axis indicates the XCI ratio (eg. S11 XCI ratio by Hum = 75∶25). XCI ratio estimated by fitting allele ratios to the beta distribution can provide information about parental bias in XCI ratio as in the patient (S18) has 82.7∶20.3 biased XCI that favors the expression of Xm (magenta). The ratio of allele expression from the maternal chromosome to the allele expression from the paternal chromosome (blue) gives the XCI ratio. In S18, using the beta model, we were able to determine that moderately skewed XCI ratio favored the expression of Xm compared to Xp. We had no phase information on the AR locus for the HUMARA assay, thus phase of XCI could not be determined. Due to homozygosity at the AR locus, S34 was uninformative for HUMARA, underlying the utility of RNAseq in XCI estimation. The SP method does not consider allele phase to estimate the parameters of allele distributions, so phase of XCI could not be determined. RNAseq estimates random XCI (<80∶20) in S14 and S23 compared to moderately skewed XCI (>80∶20) by HUMAR. S18 and S11 show complete concordance between the three methods. There is no clear trend that would indicate a higher likelihood of biased inactivation of either parental chromosome. (TIF) [file pone.0113036.s002.tif]

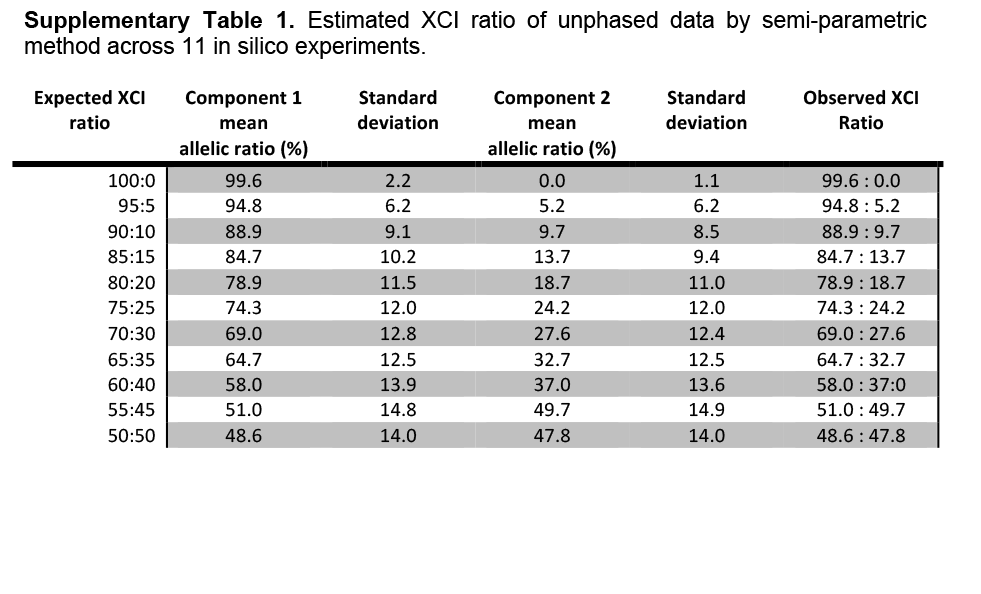

Supplement: S1 Table — Estimated XCI ratio of unphased data by SP method across 11 in silico experiments. (TIF) [file pone.0113036.s003.tif]

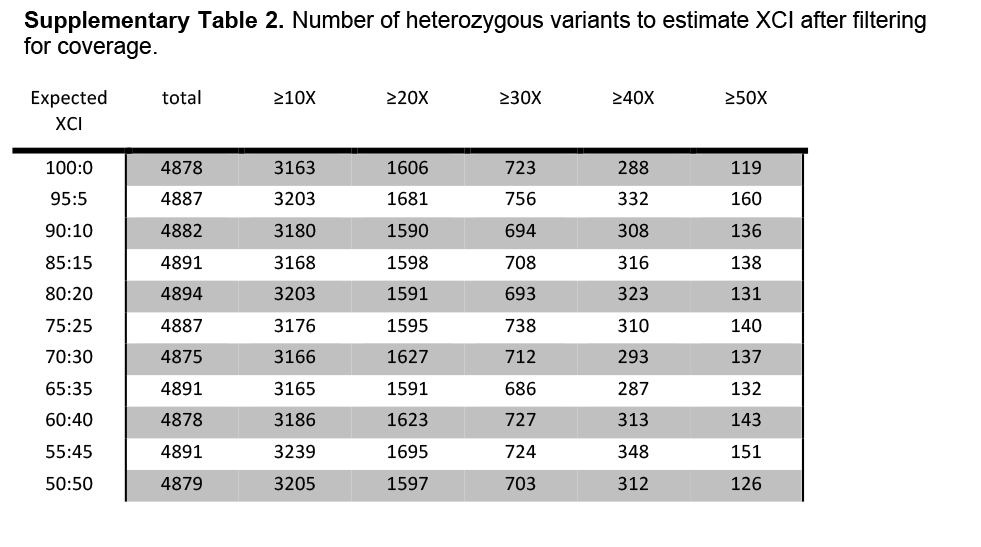

Supplement: S2 Table — Number of heterozygous variants to estimate XCI after filtering for coverage. (TIF) [file pone.0113036.s004.tif]

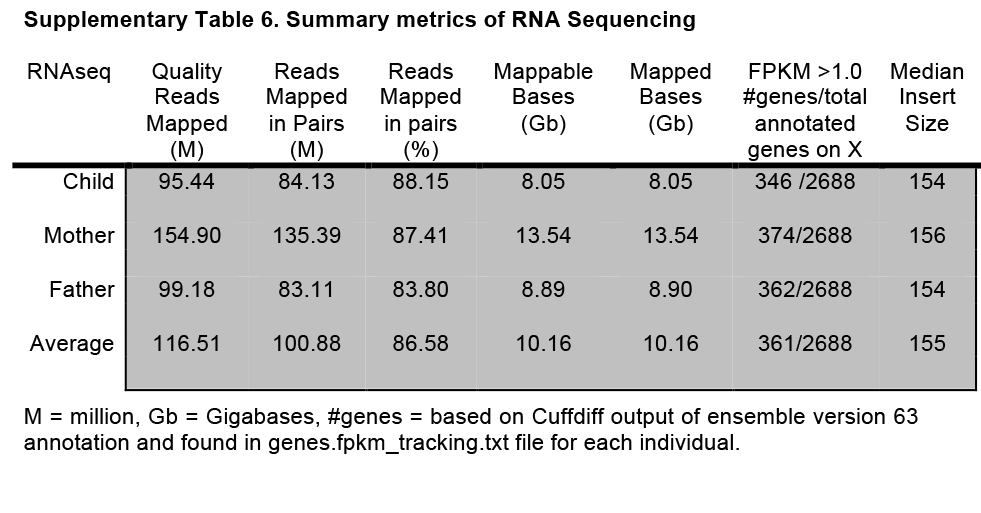

Supplement: S6 Table — Summary metrics of RNA Sequencing. (TIF) [file pone.0113036.s008.tif]
